# Target detection of helicopter electric power inspection based on the feature embedding convolution model

**DOI:** 10.1371/journal.pone.0311278

**Published:** 2024-10-07

**Authors:** Dakun Liu, Wei Zhou, Linzhen Zhou, Wen Guan

**Affiliations:** School of Mechanical Engineering, Yancheng Institute of Technology, Yancheng, Jiangsu Province, P. R. China; Xi’an Jiaotong University, CHINA

## Abstract

This study aims to improve the helicopter electric power inspection process by using the feature embedding convolution (FEC) model to solve the problems of small scope and poor real-time inspection. First, simulation experiments and model analysis determine the keyframe and flight trajectory. Second, an improved FEC model is proposed, extracting features from aerial images in large ranges in real time and accurately identifying and classifying electric power inspection targets. In the simulation experiment, the accuracy of the model in electric power circuit and equipment detection is improved by 30% compared with the traditional algorithm, and the inspection range is expanded by 26%. In addition, this study further optimizes the model with reinforcement learning technology, conducts a comparative analysis of different flight environments and facilities, and reveals the diversity and complexity of inspection objectives. The performance of the optimized model in fault detection is increased by more than 36%. In conclusion, the proposed model improves the accuracy and scope of inspection, provides a more scientific strategy for electric power inspection, and ensures inspection efficiency.

## Introduction

With the rapid development of the power industry, electric power inspection, as a crucial means to ensure the safe operation of power lines and equipment, plays a vital role in preventive maintenance and fault troubleshooting. Helicopter electric power inspection, as an innovative inspection method, leverages the high-altitude perspective and rapid flight speed of helicopters, offering advantages such as wide coverage, fast inspection speed, and high accuracy [1]. However, despite the numerous advantages of helicopter electric power inspection, target detection still has some challenges. Traditional methods heavily rely on manual inspection, where personnel visually observe from helicopters, leading to limitations in inspection scope and poor real-time capabilities [2]. Due to the substantial human and time resources required for manual inspection, it struggles to meet the demands of modern electric power inspection. Additionally, manual inspection is prone to subjective factors such as environmental influences and human misjudgments, challenging the accuracy and consistency of target detection [3]. Therefore, in the current field of electric power inspection, addressing the pressing issue of how to apply new computer vision technology to enhance the target detection accuracy and efficiency of helicopter electric power inspection is crucial. The introduction of a feature embedding convolution (FEC) model can facilitate the efficient detection of power equipment and lines during helicopter inspections [4]. The primary motivation of this study is to enhance the efficiency and accuracy of helicopter electric power inspection to meet the demands of modern power systems. This study aims to achieve efficient and accurate detection of power equipment and lines by introducing a FEC model. The advantage of this model lies in its ability to rapidly extract features from aerial images and accurately identify and classify targets based on their distinctive features. Combining deep learning (DL) and image processing techniques, this model swiftly extracts features from aerial images and achieves automated detection of electric power inspection targets by accurately recognizing and classifying their features. Utilizing the proposed model for target detection in helicopter electric power inspection fully leverages its advantages, thereby improving the accuracy and efficiency of inspections. Numerous studies in this field have already provided crucial technical references and theoretical support for the advancement of this study.

Rahmaniar and Hernawan (2021) proposed a human target detection model Single Shot MultiBox Detector (SSD) MobileNet V2 for image understanding in computer vision. The detection of human bodies in images could provide crucial information for various applications in intelligent systems. The study employed DL technology for human detection, which achieved remarkable success in various target detection applications [5]. Saleh et al. (2021) introduced an optimized Convolutional Neural Network (CNN) model for fake news detection. They compared this model with other machine learning models, including CNN, Long Short-Term Memory (LSTM), and six conventional machine learning techniques (decision trees, logistic regression, k-nearest neighbors, random forests, support vector machines, and naive Bayes) in terms of fake news detection performance. By constructing and comparing different machine learning and DL models, they demonstrated the superior performance of the optimized CNN model in fake news detection [6]. Yang et al. (2020) discussed power line inspection technology in the context of smart grids, covering inspection tasks, platforms, sensors, and automated inspection methods. To achieve automated power line inspection, they proposed or improved some advanced inspection methods, such as DL-based target detection, image segmentation, and fault diagnosis. These methods can enhance inspection efficiency and accuracy while reducing the possibility of human intervention and oversight [7]. Zhang et al. (2021) proposed a target detection method for helicopter electric power inspection based on multi-feature fusion. This method utilized various feature extraction methods, including texture, shape, and color features, to describe the characteristic information of power lines and equipment. The method achieved high target detection accuracy and robustness by fusing diverse features. Additionally, the method employed ensemble learning techniques to improve the model’s generalization ability and robustness [8]. Lee and Kim (2022) presented an adaptive adjustment-based target detection method for helicopter electric power inspection. This method could adjust and optimize the model to adapt to different environments and types of power facilities. The method utilized online learning technology to perform real-time updates and optimizations, enhancing the model’s accuracy and robustness. Moreover, the method used a hybrid loss function that considered both classification loss and localization loss, improving the precision and stability of target detection [9]. Chen et al. (2023) pointed out that most in-painting algorithms often face issues such as image blurring, texture distortion, and semantic inaccuracies. The in-painting effect was limited, especially for images with large missing areas and high resolution. To address these problems, they proposed an improved two-stage in-painting network based on parallel networks and contextual attention. Firstly, an improved deep residual network was utilized to generate pixel filling for missing areas, and the first-level adversarial network completed edge information filling. Secondly, the color features of the filled image were extracted, and the edge map was fused and supplemented, serving as the conditional label for the second-stage adversarial network. Finally, image restoration results were obtained through the two-stage network with a context attention module. Experiments on public datasets demonstrated that the proposed algorithm achieved more realistic restoration effects [10]. Han et al. (2022) proposed a fault diagnosis method based on deep migration CNN to solve the problem of insufficient training data in target domain. Transfer learning was used to learn rich feature representation from source domain, and over-fitting was reduced by global average pooling. Experiments showed that this method performed well under a small number of label samples, which provided a new idea for intelligent monitoring and diagnosis of mechanical systems [11]. To sum up, there are obvious shortcomings in the research of helicopter power inspection at present. Although previous studies mainly focused on traditional feature extraction and classification methods, they ignored the importance of multi-level and multi-modal feature fusion and deep semantic understanding. These limitations lead to the performance degradation and poor robustness of target detection algorithms in complex backgrounds and different environments. In addition, the existing methods often ignore the key requirements of real-time processing and efficiency, thus limiting their practical application. In order to make up for these research gaps, a novel and innovative target detection method is proposed in this study, which is based on the advanced FEC model. The proposed method not only uses CNN for feature extraction, but also incorporates fine feature embedding technology. This combination significantly enhances the robustness and generalization ability of the extracted features, thus achieving more accurate and reliable target detection. In addition, this study also creatively integrates the multi-modal feature fusion strategy. By effectively integrating visual and semantic information, FEC model realizes deep semantic understanding of power lines and equipment. This enhanced understanding not only improves the detection accuracy, but also enhances the adaptability of the model to different and complex scenes. More importantly, the FEC model has been carefully trained and optimized to balance accuracy and efficiency. Through improved loss function and advanced optimization algorithm, the model can achieve excellent performance while maintaining real-time processing ability. This makes FEC model especially suitable for the practical application of helicopter power inspection, in which accuracy and efficiency are very important.

The purpose of this study is to improve the intelligent level of helicopter power inspection by artificial intelligence (AI) technology to improve the overall efficiency of this detection method. Firstly, this study constructs a target detection model suitable for helicopter power inspection by analyzing the characteristics of aerial images and power lines and equipment. The model uses CNN to extract features, and combines feature embedding technology to improve the robustness and generalization ability of features. Then, the improved LSTM algorithm is used to train and optimize the model to improve the accuracy and efficiency of target detection. Finally, the effectiveness and superiority of the algorithm are verified by a large number of simulations and field experiments. In addition, the model is optimized and improved to meet the needs of target detection in different environments and power facilities. This study shows remarkable innovation in the field of helicopter power inspection. The research team successfully built a target detection model based on artificial intelligence, and optimized it with LSTM algorithm, which greatly improved the efficiency and accuracy of inspection. In addition, the experiment verifies its reliability in practical application, which contributes innovative ideas and methods for the intelligent development of power industry. The main contributions of this study include: (1) Introduce AI helicopter power inspection, improve intelligence and efficiency, solve the limitations of existing inspection, and pursue high efficiency and accuracy. (2) Build a helicopter inspection target detection model, and use CNN and feature embedding technology to detect power equipment efficiently and accurately. (3) The improved LSTM algorithm is adopted to optimize the model to improve the detection accuracy and efficiency and meet the requirements of power inspection. (4) Simulation and field experiments verify the effectiveness of the algorithm, and ensure that it is reliable and efficient in practical application.

## Target detection method based on the CNN model

This study proposes a target detection method based on the FEC model to improve the accuracy and robustness of target detection. The method uses CNN to extract image features and combines feature embedding technology to fuse and optimize the features. Firstly, a multi-scale CNN model is constructed, which is composed of multiple convolution layers of different scales, and the features of images at diverse scales can be extracted simultaneously. In this way, various details of the target object can be better captured, and the accuracy of detection can be improved. Secondly, feature embedding technology is introduced to fuse features of various scales. By splicing, fusing, and reclassifying features of different scales, the feature information at different scales can be fully utilized to improve the robustness of target detection. At the same time, an adaptive weight strategy is used to dynamically adjust the weights of each scale feature according to the importance of different scale features to target detection, thus optimizing the model’s performance. In the experiment, the commonly used target detection data set is used for verification, and the proposed method is compared with the existing target detection methods.

**Fig 1:**
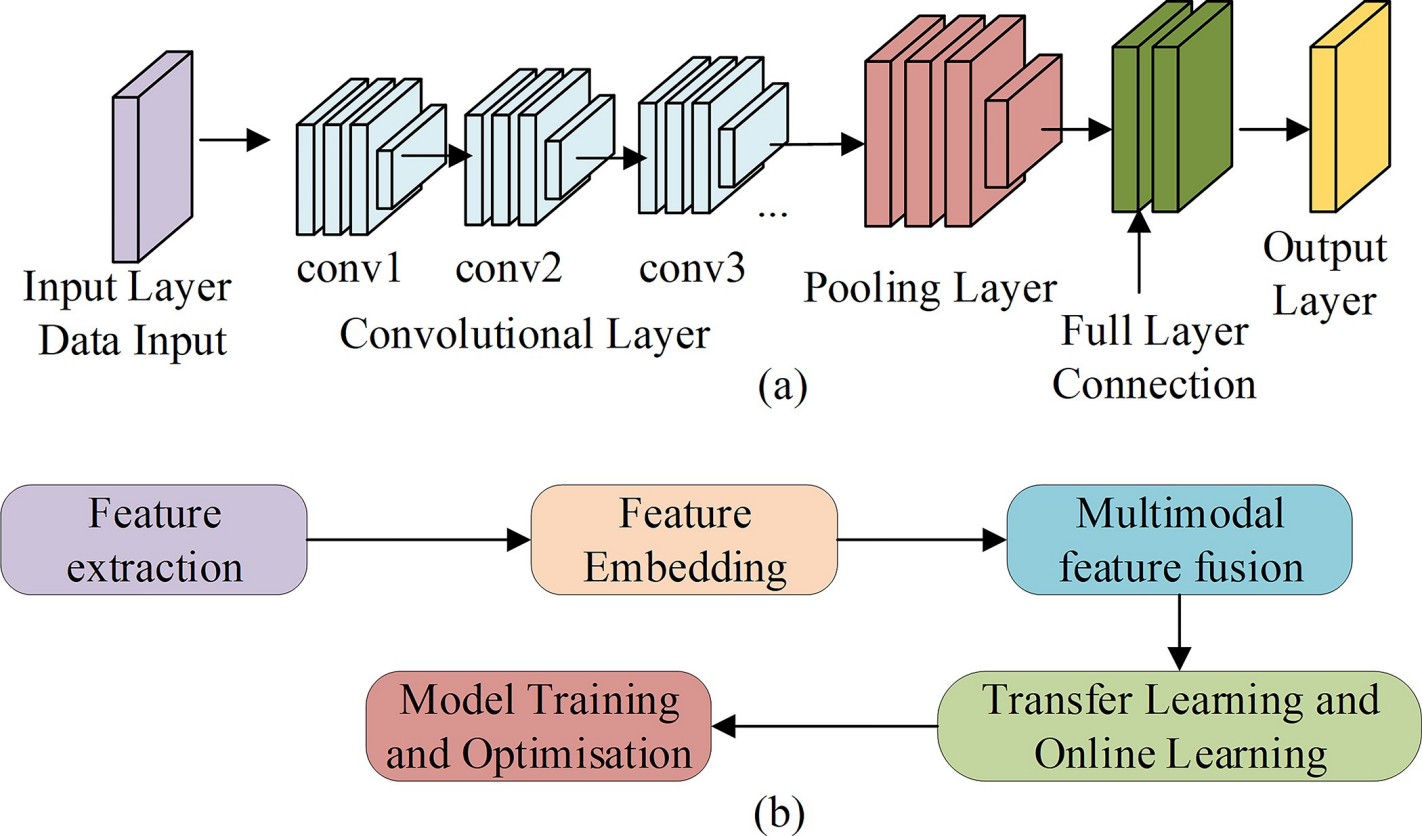
The basic structure and design flow of the FEC model (a: The basic structure; b: The design flow).

The FEC model-based target detection method is a DL model that aims to extract the feature information related to the target from the input image and classify and locate the target. The design of this model mainly includes feature extraction, feature embedding, multi-modal feature fusion, model training and optimization, transfer learning, and online learning [12]. The FEC model’s basic structure and design flow are displayed in Fig 1.

Feature extraction is one of the key steps in target detection, which aims to extract feature information related to the target from the input image. This study uses CNN to extract features in the target detection method based on the FEC model. CNN is a DL model, which can automatically extract feature expressions from images through learning [13]. In the feature extraction stage, the input image is propagated forward through CNN to get the feature map. To improve features’ robustness and generalization ability, this study introduces feature-embedded technology into target detection [14]. Feature embedding is a technique to map high-dimensional feature vectors to low-dimensional space, which can improve target detection performance by retaining important feature information and removing noise and redundant information [15]. In this study, the idea of online learning is adopted, and each type of power equipment and line is regarded as an independent category, and a separate CNN is used for feature extraction and embedded [16].

The shape characteristics of power lines and power equipment include visual information such as texture, shape, and color of images and semantic information such as the type and state of equipment. In order to make full use of visual information and semantic information for target detection, this study adopts the multi-modal feature fusion method [17]. This method aims to effectively fuse different modes’ features to obtain richer feature expressions. This study adopts a multimodal feature fusion method based on an attention mechanism. This method realizes the effective fusion of different features by learning the weight relationship among different modal features [18]. The specific implementation process is as follows. First, the visual and semantic features are input into two independent CNNs to get their respective attention weights. Second, the semantic and visual features are fused according to their respective weights, and the final feature representation is obtained [19]. The typical structure of CNN usually encompasses an input, convolution, pooling, fully connected, and output layer. For the input data, CNN extracts its features [20]. The equation of CNN for feature extraction of data reads:

$$H_{i}=f(W_{i⊗}H_{i-1}+b_{i})$$ (1)

***i*** represents the convolution level of the network. **W** refers to the calculation weight, and **b** means the offset vector in the calculation process. Through the excitation of the excitation function, the feature vector **H**_***i***_ is obtained. The calculation of the pooling process of CNN is:

$$H_{i}=subsampling(H_{i-1})$$ (2)

After multiple pools, the changed features are represented and classified through a fully connected network, and the final mapping result is shown in Eq (3):

$$Y(m)=P(L=l_{m}|H_{0};(W,b))$$ (3)

***m*** stands for the index of tag category. **L** indicates loss function, and ***P*** refers to mapping operation. The expression of loss function is as follows:

$$NLL(W,b)=-\sum_{m=1}^{\left|Y\right|}log\;Y(m)$$ (4)

MSE(W,b)=1|Y|∑m=1|Y|(Y(m)−Y^(m))2 (5)

To reduce the over-fitting of network parameters, a two-norm term is usually added to the final loss function, and its calculation is:

$$E(W,b)=L(W,b)+\frac{\lambda }{2}W^{T}W$$ (6)

$$W_{i}=W_{i}-\eta \frac{\partial E(W,b)}{\partial W_{i}}$$ (7)

$$b_{i}=b_{i}-\eta \frac{\partial E(W,b)}{\partial b_{i}}$$ (8)

***η*** represents the learning rate [21]. The optimization design of the CNN model based on LSTM is mainly aimed at optimizing the combination mode and parameters of CNN and LSTM to improve the model’s performance and generalization ability [22].

The method adopted in this study is a multi-modal fusion strategy based on an attention mechanism. Multi-modal fusion refers to the fusion of different modal data to obtain richer feature representation [23]. In the CNN model based on LSTM, a multi-modal fusion strategy can be used to fuse visual features and semantic features to obtain more accurate prediction results [24]. A multi-modal fusion strategy based on an attention mechanism means that in the fusion process, the important information in different modal data is automatically paid attention to through the attention mechanism, and weighted fusion is carried out. The specific implementation method is as follows. More accurate prediction results can be obtained by introducing attention mechanisms between visual and semantic features and by calculating attention weights to fuse features from different modes [25]. By adopting the attention mechanism-based multi-modal fusion strategy, the comprehensive understanding ability of the model to different modal data can be improved, and the model’s prediction accuracy and generalization ability can be enhanced [26]. Meanwhile, this method can also be extended to other multimodal tasks, such as speech recognition, natural language processing, and so on [27]. Eq (9) is the calculation of attention weight.

$$W=softmax(W_{a}*[V,T])$$ (9)

***W***_***a***_ represents attention weight matrix. ***V*** refers to visual feature matrix. ***T*** is semantic feature matrix, and ***softmax*** function is used to calculate attention weight.


W′=softmax(concat(V,T)*tanh(concat(V,T))) (10)

***W*′**is the fusion weight. ***concat*** means splicing two matrices, and ***tanh*** indicates hyperbolic tangent activation function. The expression of the fusion result reads:

$$W^{\prime \prime }=V*W\prime +T*(1-W\prime )$$ (11)

***W***″ is the fusion result. In the data preprocessing stage, it is usually necessary to denoise and normalize the data to ensure the accuracy and reliability of the data [28]. Specifically, the following equation is used to preprocess the data:

$$x^{\prime }=(x-min(x))/(max(x)-min(x))$$ (12)

***x*** is the original data and ***x***′ is the normalized data [29]. The prediction equation is as follows:

$$y_{\left(t+1\right)}=f(w_{0}+h(w_{1}*x_{\left(t\right)}+w_{2}*x_{\left(t-1\right)}+\ldots +w_{n}*x_{\left(t-n\right)}))$$ (13)

***y***_(***t***+**1**)_ signifies the prediction result. ***x***_(***t***)_ denotes the input data. ***w*** is the weight coefficient of the neural network. ***n*** indicates the order of the neural network. ***f*** represents the activation function, and ***h*** means the network output function [30]. Optical influence analysis is a technique to analyze the interaction between parameters, and its equation can be expressed as:

$$y_{i}=\beta_{i}+\beta_{1}x_{i1}+\beta_{2}x_{i2}+\cdots +\beta_{k}x_{ik}+\varepsilon_{i}$$ (14)

***y***_***i***_ indicates the target parameter. ***x***_***i***_ represents the independent variable. ***β*** denotes the autoregressive coefficient. ***ε***_***i***_ is the error term, and ***k*** means the number of independent variables. Through the prediction and optical influence analysis, the prediction results of each parameter data and the interaction model can be obtained, and the best operation strategy can be selected based on this, and its expression can be written as:

O=argmax(f(y1,y2,…,yn)) (15)

**Fig 2:**
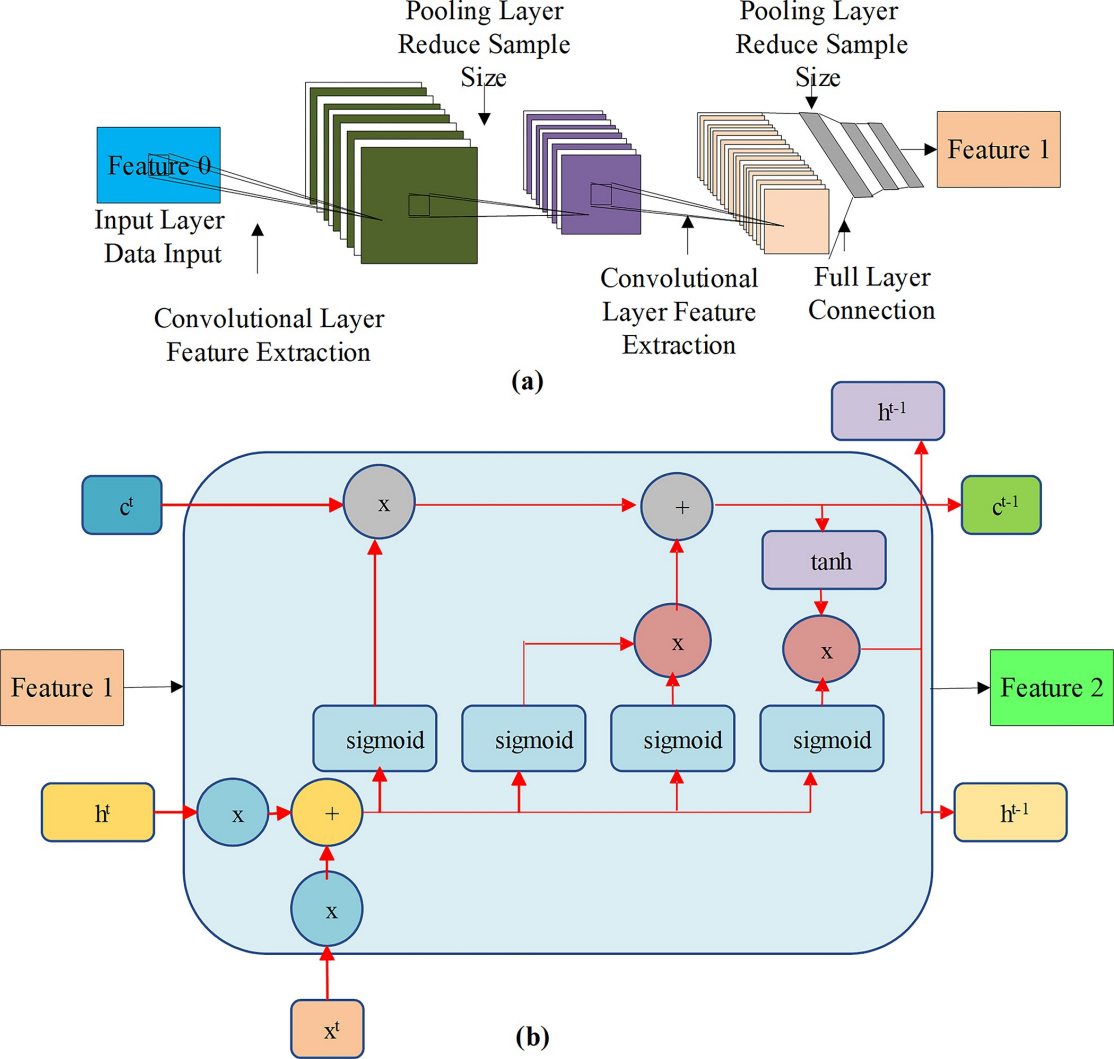
Structure diagram of optimal design of the FEC model (a: The calculation structure of the CNN model; b: The calculation structure of the LSTM model).

***y*** is the prediction result of each parameter data; ***f*** is the optimization function of operation strategy. The target function is usually to minimize energy-saving cost or maximize energy-saving benefit [31]. Through the above optimization, this study integrates the CNN model with the LSTM model, thus realizing the optimization of the FEC model, and thus designing the FEC-LSTM [32]. Fig 2 suggests the optimization structure of the FEC model through the LSTM model.

**Fig 3:**
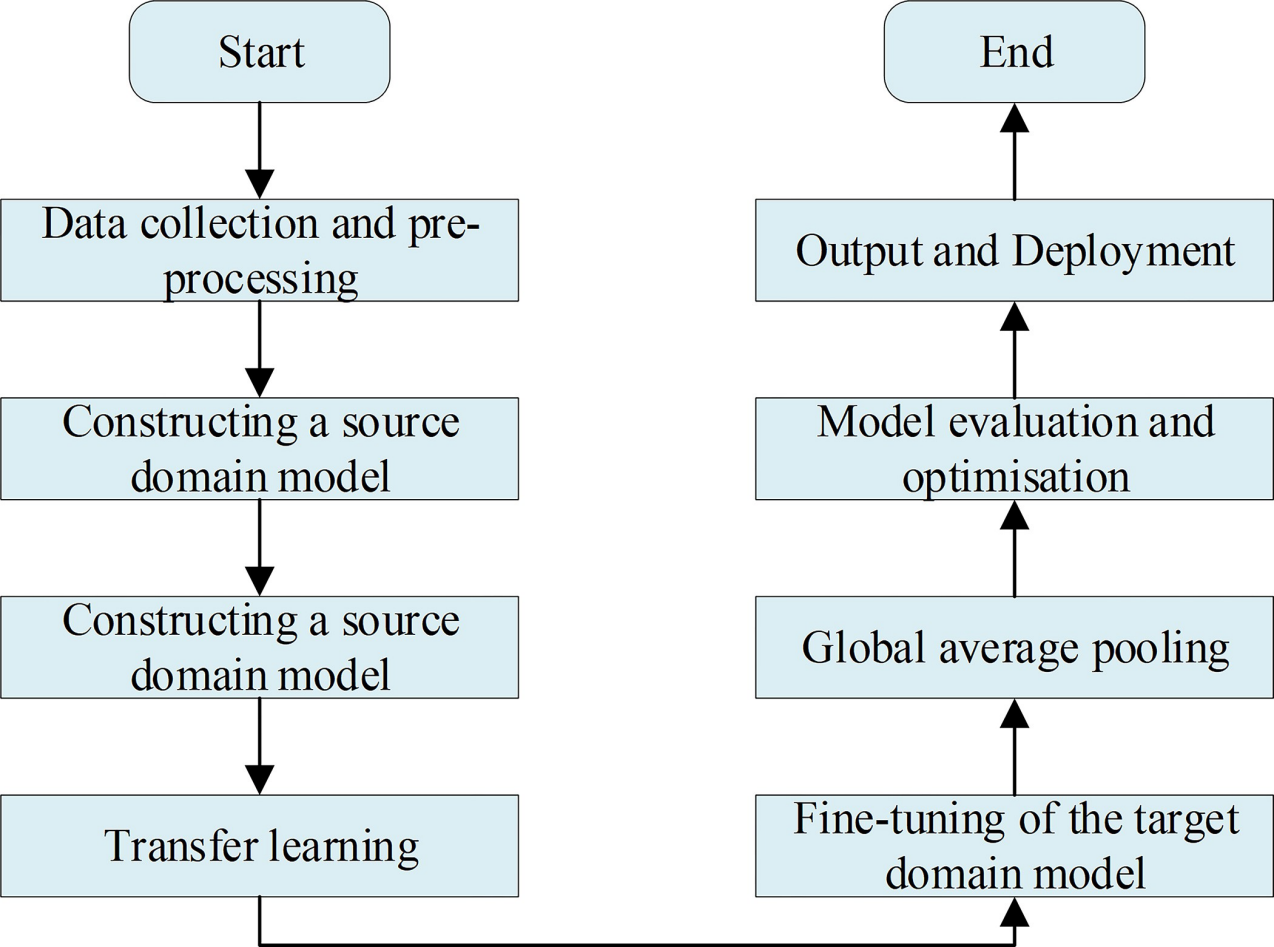
Calculation flow chart.

Fig 2 denotes that this study initially constructs a basic FEC model. This model employs convolutional layers to perform convolution and pooling operations on input data iteratively, extracting feature information from the images. Subsequently, the CNN model’s output features serve as the LSTM model’s input features. The LSTM model, a type of recurrent neural network, can handle sequential data and capture temporal dependencies. By embedding the features extracted by the CNN into the LSTM, the model leverages the memory capabilities of LSTM to capture long-term dependencies within the sequence while utilizing the features extracted by the CNN for prediction. This combined approach effectively uses the strengths of both models, such as the feature extraction capability of the CNN and the sequential modeling ability of the LSTM, resulting in more accurate and efficient predictions [33]. Table 1 and Fig 3 show the algorithm design and calculation flow chart of the proposed model.

**Table 1:** Algorithm design.

Step	Description	Code
1	Import necessary libraries	import numpy as np<br>from keras.models import Sequential<br>from keras.layers import Conv2D, MaxPooling2D, Flatten, Dense, LSTM
2	Define function to create Convolutional Neural Network (CNN) model	def create_convolutional_model(input_shape):<br> model = Sequential()<br> model.add(Conv2D(32, kernel_size = (3, 3), activation = ’relu’, input_shape = input_shape))<br> model.add(MaxPooling2D(pool_size = (2, 2)))<br> model.add(Conv2D(64, kernel_size = (3, 3), activation = ’relu’))<br> model.add(MaxPooling2D(pool_size = (2, 2)))<br> model.add(Flatten())<br> model.add(Dense(128, activation = ’relu’))<br> return model
3	Define function to create Long Short-Term Memory (LSTM) network model	def create_lstm_network(input_shape):<br> model = Sequential()<br> model.add(LSTM(128, input_shape = input_shape))<br> model.add(Dense(1, activation = ’sigmoid’))<br> return model
4	Create CNN model for feature extraction	cnn_model = create_convolutional_model((32, 32, 3))
5	Create LSTM network model	lstm_model = create_lstm_network((None, 128))
6	Use extracted features from CNN as input to LSTM	cnn_features = cnn_model.predict(X_train)<br>cnn_features = cnn_features.reshape((cnn_features.shape[0], 1, cnn_features.shape[1]))
7	Combine CNN and LSTM as part of overall model	final_model = Sequential()<br>final_model.add(lstm_model)<br>final_model.add(Dense(num_classes, activation = ’softmax’))
8	Compile and train the model	final_model.compile(loss = ’categorical_crossentropy’, optimizer = ’adam’, metrics = [’accuracy’])<br>final_model.fit(cnn_features, y_train, epochs = 10, batch_size = 32)
9	Evaluate the model	test_features = cnn_model.predict(X_test)<br>test_features = test_features.reshape((test_features.shape[0], 1, test_features.shape[1]))<br>test_loss, test_accuracy = final_model.evaluate(test_features, y_test)<br>print("Test loss:", test_loss)<br>print("Test accuracy:", test_accuracy)

Table 1 shows in detail the steps of creating a mixed model of convolutional neural network and long-term and short-term memory network using Keras. This hybrid model combines the advantages of CNN and LSTM, and is suitable for processing data with both spatial and time series characteristics, such as video classification or prediction from image sequences. Firstly, import the necessary libraries, including ’numpy’ for numerical operation and ’keras’ module for constructing neural network. The first function `create _ voluntary _ model (input _ shape) `defines a CNN model. The model starts with a’ Sequential’ container, and adds a convolution layer (’Conv2D’) and a maximum pooling layer (’MaxPooling2D’) in turn. The convolution layer is responsible for extracting spatial features from the input image, while the pooling layer reduces the spatial dimension and retains the most significant features. The final output is flattened and passed through a fully connected layer (`Dense`) to generate a feature vector. The second function `create _ LSTM _ network (input _ shape)`defines an LSTM model. LSTM is very powerful in capturing the time dependence of sequence data. This function initializes a Sequential model, adds an LSTM layer, and then adds a fully connected layer with sigmoid activation function, which is suitable for binary classification tasks. After defining the model, the CNN model is instantiated with the input data in the shape of (32, 32, 3), which represents an image with 3232 pixels and three-color channels. CNN model processes training data to extract features. These features are readjusted to meet the input requirements of the LSTM model, that is, the expected input sequence. Then initialize a new sequence model to combine CNN and LSTM models. The LSTM model with CNN features is added to this new model, and finally a fully connected layer with softmax activation function is added to make the model suitable for multi-classification tasks. The model is compiled by using `categorical_crossentropy’ loss function, `adam’ optimizer and accuracy as indicators. The training process is started by calling the `fit`method, and the model learns from the training data. After the training is completed, the performance of the model is evaluated by using the test data, and the test loss and test accuracy are printed. This hybrid model method has obvious advantages because it combines the feature extraction ability of CNN and the sequence modeling ability of LSTM. This method is very useful especially in applications that need to analyze spatial and temporal data at the same time. For example, in video analysis, CNN can extract spatial features from a single frame, while LSTM can model the temporal relationship between these frames. Although the integration process is relatively simple in this example, it can adapt to more complex scenarios and provide flexibility in model design and application.

## Model evaluation experiment design

This study comprehensively evaluates the designed model through training and test evaluation, in which training and test evaluation mainly depend on datasets. Here, this study adopts the open IEEE 14-node power system test case as the experimental dataset, which is a 14-node power system model, covering the data of key components such as generators, transmission lines and loads. This dataset provides generator parameters associated with each node in detail, including instantaneous current, generator capacity, generator type (such as generator or synchronous motor), steady state and speed, etc. In addition, the dataset also contains the transmission line parameters of the connecting nodes, including the impedance, admittance and capacity of the line. IEEE 14-node system datasets are widely used in the research of power system stability, dynamic response and control. Researchers can use this dataset to simulate, analyze, develop and verify various power system algorithms and control strategies. Through the analysis of IEEE 14-node system, researchers can evaluate the stability, short-circuit ability and power transmission ability of power system, and design corresponding control schemes to improve the performance and reliability of power system. In order to further verify the performance of the model, this study also combines the existing foreign body datasets of transmission lines and infrared and visible image datasets of power lines to carry out simulation experimental evaluation. The selection of these datasets aims to fully reflect the diversity and complexity of real-world power detection scenarios, including the status of transmission lines under different weather conditions, various foreign bodies that may appear on transmission lines, and power line images under different lighting conditions. By comprehensively using these datasets, this study can comprehensively explore the comprehensive performance of the model in power detection and provide important support for the development of power detection. These simulation experiments not only verify the accuracy and reliability of the model, but also provide strong data support for subsequent research and improvement. The environment of this experiment is: under Win7 operating system, the processor is Intel Xeon CPU E5-4607V2 @ 2.60 GHz, with 6 cores, 12 logics, 32 GB of RAM and 2012b of MATLAB. Table 2 shows the design results of model parameters.

**Table 2:** Model parameter design.

Layer type	Input shape	Output shape	Parameters/configuration
Input layer	(32, 64, 64, 1)	(32, 64, 64, 1)	--
Convolution layer 1	(32, 64, 64, 1)	(32, 64, 64, 32)	Convolution kernel size: (3, 3, 3), step size = 1, padding = 0
Convolution layer 2	(32, 64, 64, 32)	(32, 64, 64, 64)	Convolution kernel size: (3, 3, 3), step size = 1, padding = 0
Pool layer 1	(32, 64, 64, 64)	(32, 64, 64, 64)	Window size: (2, 2, 2), step size = 2
Pool layer 2	(32, 64, 64, 64)	(32, 64, 64, 64)	Window size: (2, 2, 2), step size = 2
Fully connected layer	(32, 64, 64, 64)	(100, 5)	Activation function: ReLU
Output layer	(100, 5)	(100, 5)	--

Firstly, this study compares and evaluates the designed basic FEC model with more advanced technological models, including Liu et al.’s (2023) Infrared Small and Dim Target Detection Transformer (IRSTransformer) model [34]; Li and Shen’s (2023) YOLOSR-IST You Only Look Once Super Resolution-Super-Resolution (YOLOSR-IST) model [35]; Yin et al.’s (2023) Semisupervised-Synthetic Aperture Radar Ship Detection (YOLOV4_CPSBi) model [36]; Du et al.’s (2023) SS- Semisupervised-Synthetic Aperture Radar Ship Detection (SARShipDet) model [37]; Zhu et al.’s (2023) generalized likelihood ratio test (GLRT) model [38]; Khedr et al.’s (2023) energy-aware radial clustering- optimized deep convolutive learning (EARC-ODCL) model [39]. Secondly, the FEC-LSTM model designed here undergoes further comparative evaluation against the basic model and these more advanced technological models. This comprehensive assessment aims to evaluate the designed model’s performance thoroughly, thus exploring the overall value of this study.

## Performance evaluation of helicopter electric power inspection based on the FEC model

### Basic performance evaluation of the FEC model

**Fig 4:**
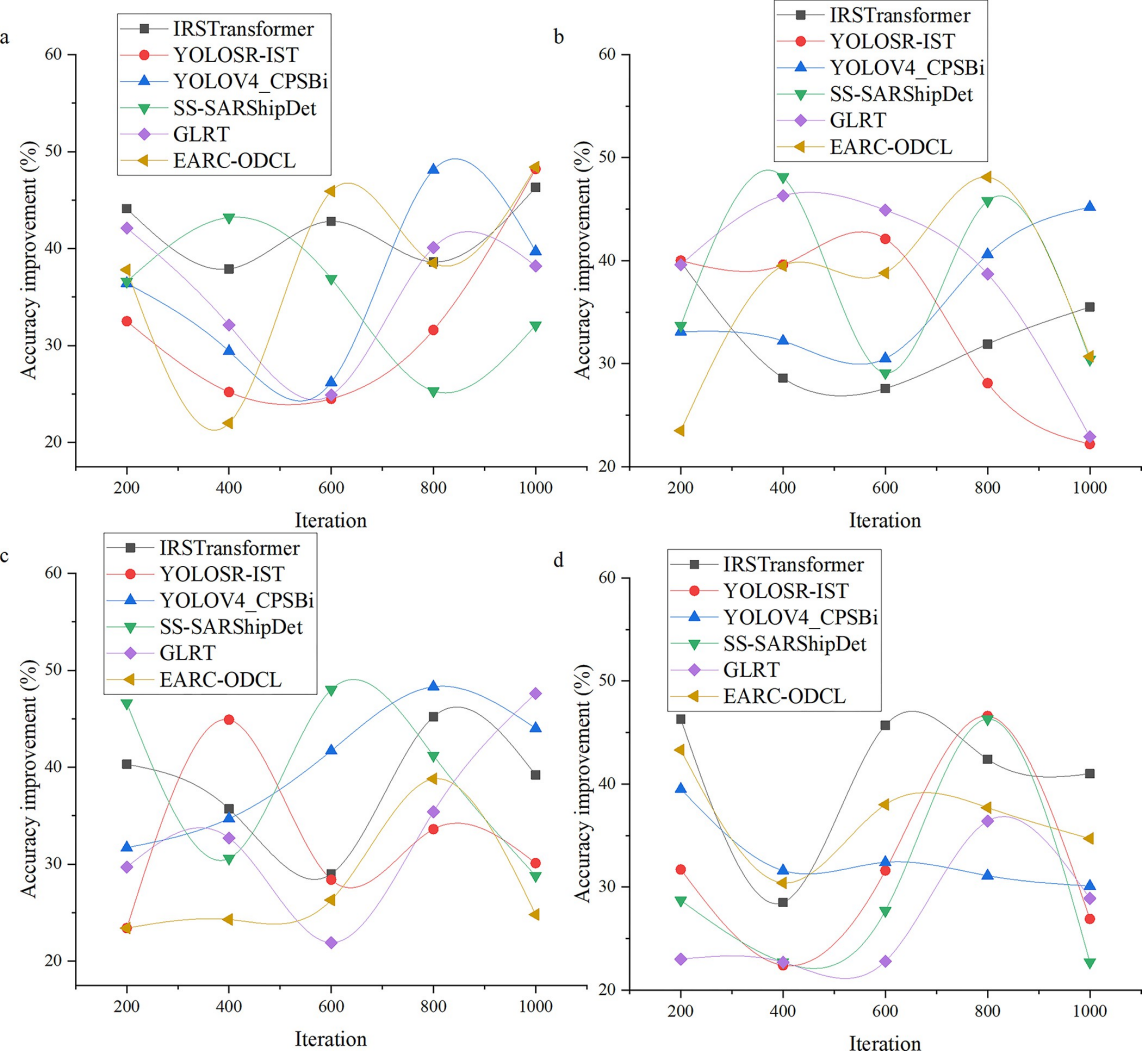
Evaluation of inspection accuracy of the FEC model (a: Generator test, b: Transmission line test, c: Load test, d: Transformer test).

To improve the performance of helicopter electric power inspection, this study first designs a FEC model, which is based on CNN model to realize feature embedded operation, thus improving the feature detection effect of the model. Based on this, to highlight the performance of the model designed in this study, this study compares and evaluates the designed FEC model with the more advanced neural network model. By comparing with the above six models, the efficiency improvement results of FEC-LATM are calculated, and the corresponding data map is drawn through the result data. The specific results of the comparative evaluation of this model are revealed in Fig 4.

**Fig 5:**
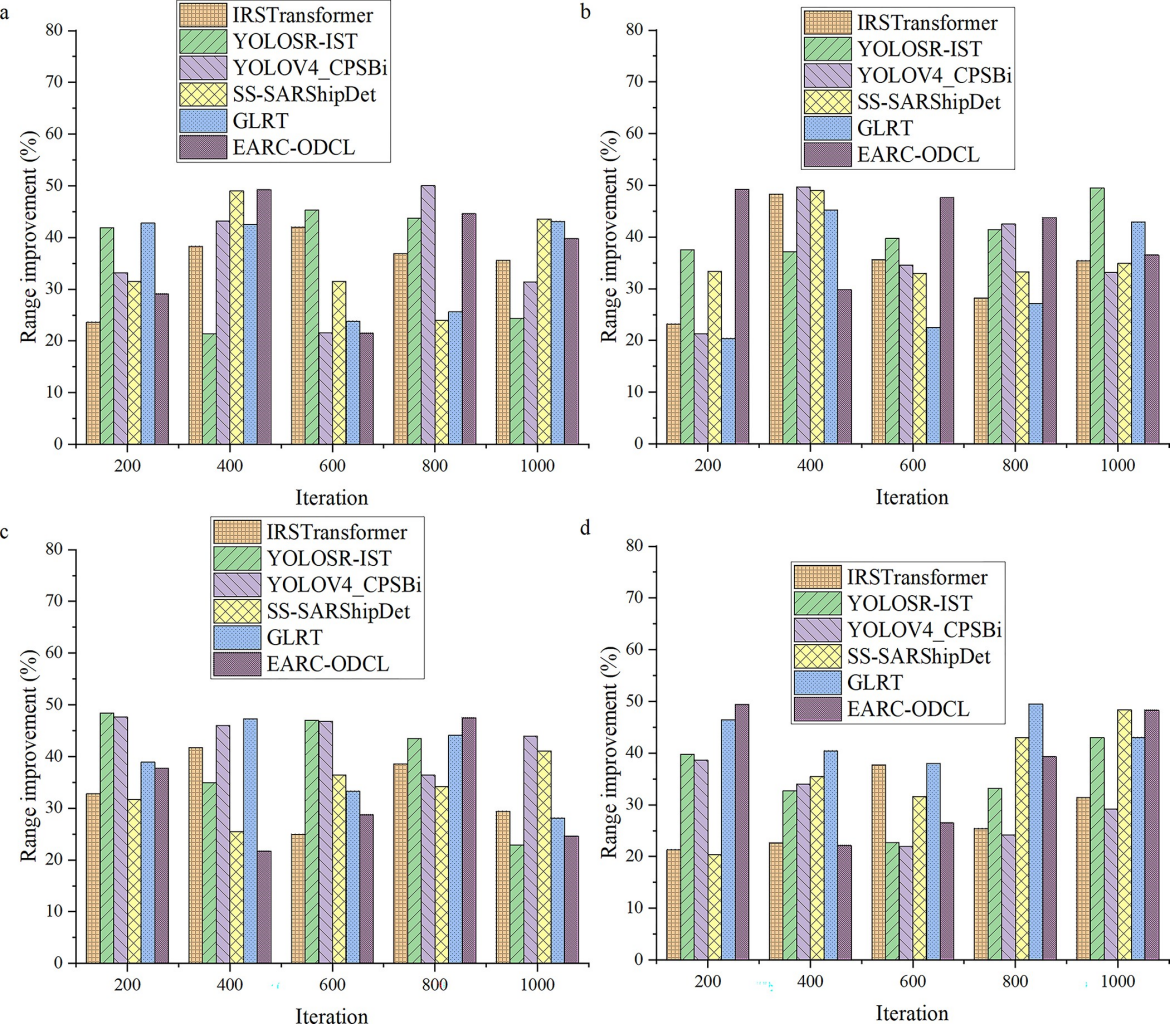
Evaluation of inspection range of the FEC model (a, b, c, and d refer to generator test, transmission line test, load test, and transformer test).

According to the experimental results in Fig 4, the FEC model designed in this study shows a significant performance improvement compared with the advanced technology model. In the detection and evaluation of various key power components in the dataset, the detection accuracy of FEC model exceeds 21%, and the average promotion rate exceeds 30%. These results strongly support the superiority of the FEC model described in the methodology section. The model captures the feature information of power components in detail, and uses advanced feature embedding technology to represent and analyze the data. By effectively integrating the features of different levels and scales, the designed model shows higher accuracy and stability in the detection task. In a word, compared with the advanced technology model, the designed FEC model has been proved to significantly improve the performance in detecting key power components. Fig 5 shows the evaluation results of the model inspection scope in this study.

Based on the experimental results shown in Fig 5, the FEC model designed in this study shows remarkable performance improvement in the evaluation of the detection range of the model. Compared with many advanced neural network models, FEC model not only breaks through the 20% barrier in detection range, but also jumps to more than 26% on average. This data not only reflects the remarkable leap in the performance of the model, but also indicates another milestone in the technical progress in the field of electric power detection. Specifically, the FEC model realizes the efficient detection of power components of different scales and sizes through carefully designed algorithms and optimized structures. Compared with the traditional model, it can capture the subtle changes of electrical components more keenly, thus providing more comprehensive and accurate detection results. This feature makes FEC model more valuable in complex and changeable power system, and can effectively deal with various challenges and problems. In addition, the FEC model also makes full use of feature embedding technology, and deeply mines and analyzes the feature information of power components through DL and pattern recognition. The application of this technology enables the model to better understand and utilize the inherent characteristics of electrical components, and then improve the detection range and detection accuracy. This not only provides strong support for the performance optimization of the model, but also lays a solid foundation for the subsequent research and application. To sum up, the FEC model designed in this study has achieved remarkable results in the evaluation of detection range, and has obvious advantages compared with other advanced neural network models. This breakthrough not only helps to improve the overall level of power detection technology, but also provides a more reliable and efficient solution for practical engineering applications. With the continuous progress of technology and the continuous expansion of application fields, it is believed that FEC model will play a more important role in the future and contribute more to the safe and stable operation of power system.

### Performance evaluation of the optimized FEC model

**Fig 6:**
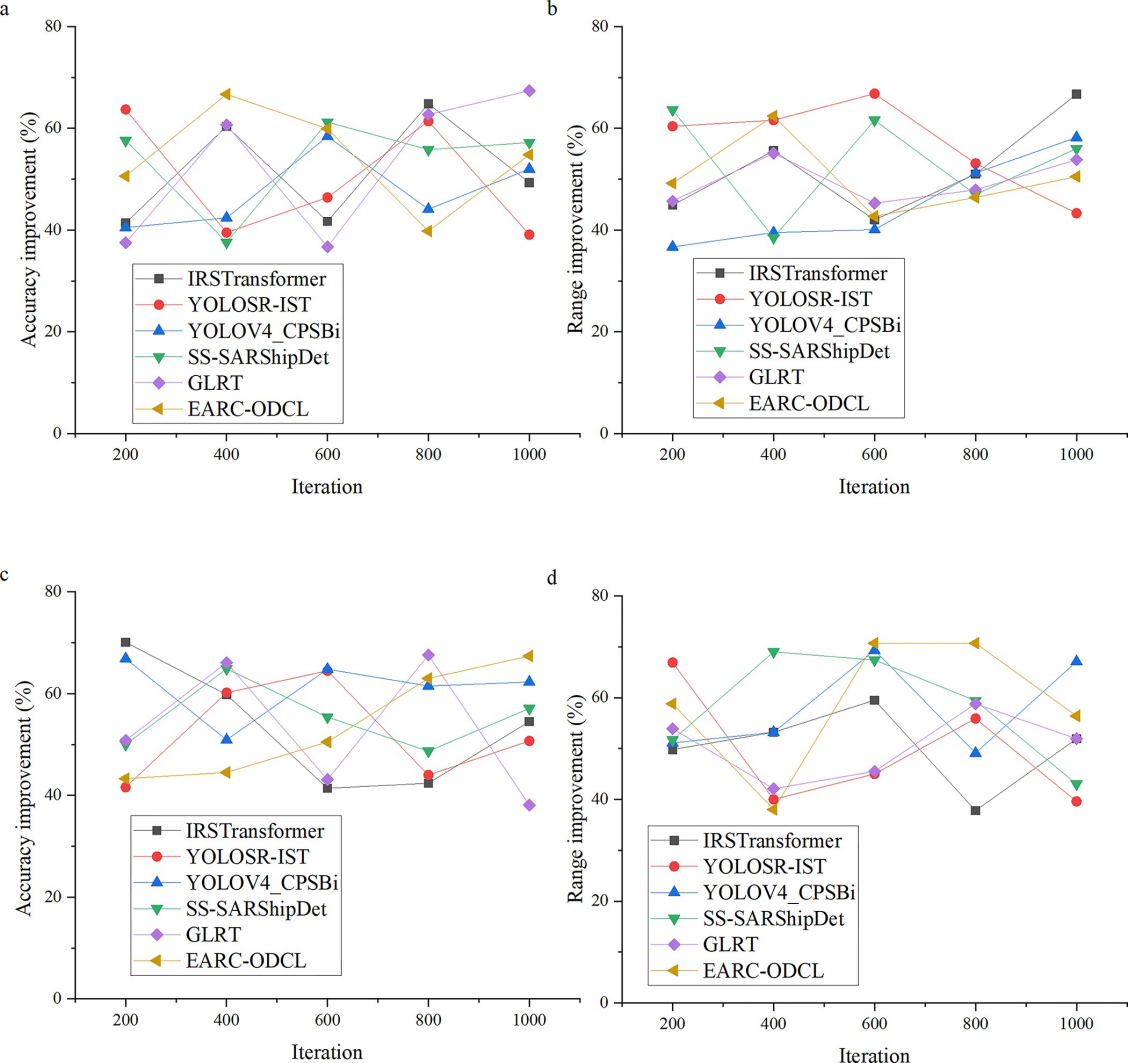
Performance evaluation of the optimized model (a: Generator inspection accuracy test, b: Generator inspection range test, c: Transmission line accuracy test, d: Transmission line range test).

On the basis of the above model evaluation, this study comprehensively optimizes it, that is, the FEC model is further strengthened by LSTM model and multimodal fusion strategy, thus improving the comprehensive performance of the model, and comprehensively evaluates the optimized model. The performance evaluation results of the optimized model are shown in Fig 6. Compared with Fig 4, in order to compare the comprehensive performance of the innovation results more clearly, the results only evaluate the effects of generator inspection and transmission line detection of the model.

The comparative evaluation results in Fig 6 show that the performance improvement rate of the integrated model designed in this study exceeds 36% in the accuracy and range test of generator and transmission line inspection. This data fully shows that the integrated model in this study has obvious advantages over other optimization models in generator and transmission line inspection tasks. By skillfully combining different technologies and algorithms, the model can identify and detect the problems on generators and transmission lines more accurately, and at the same time expand the inspection scope, which marks a key technical breakthrough in the field of power inspection. In addition, the integrated model in this study is also helpful to realize real-time helicopter power inspection and provides important technical support for the development of power industry. With the help of helicopter’s high efficiency as a tool, the model can perform power inspection tasks more quickly and comprehensively, thus improving the efficiency and accuracy of inspection. This innovation not only improves the technical level of power inspection, but also injects new vitality into the sustainable development of power industry.

## Discussion

With the rapid development of power industry, the role of power inspection in ensuring the stable operation of power system and accident prevention is becoming more and more prominent. However, the traditional power inspection method is limited by the efficiency and accuracy of inspection, and it is difficult to meet the needs of modern power system. In order to meet this challenge, a FEC model based on DL is designed by using the latest progress of AI technology, especially the excellent performance of DL in image recognition and processing, aiming at realizing efficient and accurate detection and evaluation of key electrical components in helicopter power inspection. The core of this study is to improve its performance and generalization ability by optimizing and improving the model. An innovative FEC model is proposed, which combines the advantages of CNN and LSTM network, and fuses visual and semantic features through multimodal fusion strategy. This strategy not only enriches the representation of features, but also improves the detection ability of the model for electrical components in different environments and conditions. In the process of model optimization, there are many challenges. Firstly, due to the complexity and variability of helicopter power inspection environment, the robustness and universality of the model have become key issues. In order to solve this problem, data enhancement technology is adopted. By simulating inspection images in different environments and conditions, the training data of the model is increased and its generalization ability is improved. In addition, regularization method and early stop strategy are introduced to prevent the model from over-fitting and ensure its robustness. In order to verify the performance of the model, a series of comparative evaluations were conducted. The results show that the comprehensive model designed in this study has significantly improved the accuracy and range test of generator inspection and transmission line inspection, exceeding the improvement rate of 36%. This achievement fully proves the advantages and potential of this model in helicopter power inspection. In addition, in order to further improve the real-time and efficiency of the model, this study also optimizes and adjusts the model. By optimizing the algorithm and parameter setting, the real-time and efficient execution of helicopter power inspection work is successfully realized, which provides strong technical support for the development of power industry. To sum up, the FEC model based on DL design in this study shows excellent performance and generalization ability in helicopter power inspection. By optimizing and improving the model, many challenges are successfully overcome, and the robustness and universality of the model are improved. This innovation provides an efficient and accurate solution for intelligent inspection of power industry, and injects new vitality into the sustainable development of power industry.

## Conclusion

By applying AI technology, especially the innovative FEC model, this study optimizes the process of helicopter power inspection, which significantly improves the detection performance and effect. Although a breakthrough has been made in the technical level and great practical value has been shown, there are still some limitations in this study, and the future research is prospected. Firstly, the FEC model designed in this study provides a powerful tool for power inspection, which can effectively extract the characteristics of power equipment and lines and realize accurate target identification and classification. Compared with traditional algorithms and other conventional algorithms, the accuracy of target detection is improved by more than 30%, and the detection range is expanded by more than 26%. However, the model still has some limitations in the adaptability of power inspection tasks in different types and environments. Future research can further explore cross-domain and transfer learning strategies to enhance the generalization ability of the model and make it adapt to a wider range of application scenarios. Secondly, this study optimizes the basic model based on LSTM model, which significantly improves the performance of power inspection. The improved performance of the optimized model in fault detection has reached more than 36%, which provides a strong guarantee for the accuracy and real-time performance of power inspection. However, with the continuous progress of technology, future research can consider integrating the designed model with other advanced neural network structures to further improve the efficiency and accuracy of power inspection. In addition, although this study compares the basic model and optimization model with other advanced technology models and verifies their advantages in power inspection, it still needs to pay attention to the cross-integration with other fields. Future research can explore the combination of power inspection with UAV technology and IoT technology to promote the intelligent and automated process of power inspection. Although this study has made remarkable achievements in helicopter power inspection, there are still some limitations. Future research should pay attention to the generalization ability of the model, the integration with other advanced technologies and the integration of cross-fields to promote the continuous development of power inspection technology and provide more reliable power guarantee for social development and people’s lives.

## Supporting information

S1 File (ZIP)
