# Supplementary material for: Target detection of helicopter electric power inspection based on the feature embedding convolution model
Source: PLoS One. 2024 Oct 7;19(10):e0311278. doi: 10.1371/journal.pone.0311278 (PMC11458054; doi:10.1371/journal.pone.0311278)
Supplement: S1 File — (ZIP) [file pone.0311278.s001.zip › Data packet/Dataset and description.docx]

IEEE 14-Bus system data set is a very valuable power system model, which is widely used in power system analysis and simulation research. This data set contains detailed information of 14-node power system, including data of key components such as generators, transmission lines and loads. Based on this, this paper explores the comprehensive performance of this model through the simulation experiment evaluation of the existing transmission line foreign body data set and power line infrared and visible light image data set, and provides important support for the development of power inspection work. The following is a more detailed introduction and analysis of this data set.

Each node in the data set of IEEE 14-Bus system is associated with one or more generators. These generators have different technical specifications and performance parameters, such as instantaneous current, generator capacity, generator type (such as synchronous generator or synchronous motor), steady state and speed. These parameters provide detailed information about the power generation capacity of the power system and how to optimize the performance of the power system by controlling the generators.

In addition to generator parameters, transmission line parameters associated with each node are also provided in the data set. Transmission lines are used to connect various nodes in the power system, and they have specific impedance, admittance and capacity. These parameters determine how power is transmitted in the system and how to optimize the performance of the power system by changing the characteristics of transmission lines.

The wide application of IEEE 14-Bus system data set lies in the research of power system stability, dynamic response and control. Researchers can use this data set to simulate, analyze, develop and verify various power system algorithms and control strategies. Through the analysis of IEEE 14-Bus system, researchers can evaluate the stability, short-circuit ability and power transmission ability of power system, and design corresponding control schemes to improve the performance and reliability of power system.

For example, researchers can use this data set to evaluate the impact of different control strategies on power system stability. They can observe the response of power system under different conditions by simulating different control schemes and compare the advantages and disadvantages of different schemes. In this way, researchers can design more effective control schemes to improve the stability and reliability of the power system.

In addition, IEEE 14-Bus system data set can also be used to evaluate the response of power system under fault conditions. Researchers can observe the response and stability of power system by simulating various fault situations, such as single-phase grounding fault and two-phase short circuit. In this way, they can identify the weak links and potential security risks in the power system and take corresponding measures to improve the reliability and security of the power system.

In a word, IEEE 14-Bus system data set is a very important power system model, which provides researchers with detailed data for analyzing and simulating power systems. By using this data set, researchers can better understand the behavior of power system, design and verify more effective power system algorithms and control strategies, thus improving the performance and reliability of power system. Meanwhile, this data set can also be used to evaluate the response and stability of power system under fault conditions, which provides strong support for the safety and reliability of power system.

Download URL of data set:https://link.zhihu.com/?target=https%3A//pan.baidu.com/s/1bfNcScoKfc_2nNoR_gGNIA
